# Causes and remedies for the dominant risk factors in Enterprise System implementation projects: the consultants’ perspective

**DOI:** 10.1186/s40064-016-1862-9

**Published:** 2016-02-29

**Authors:** Przemysław Lech

**Affiliations:** Faculty of Management, University of Gdańsk, ul. Armii Krajowej 101, 81-824 Sopot, Poland

**Keywords:** Enterprise System, ERP implementation, Project risk, Project failure, Consultants, Project management

## Abstract

The purpose of this research was to investigate the causes of the dominant risk factors, affecting Enterprise System implementation projects and propose remedies for those risk factors from the perspective of implementation consultants. The study used a qualitative research strategy, based on e-mail interviews, semi-structured personal interviews with consultants and participant observation during implementation projects. The main contribution of this paper is that it offers viable indications of how to mitigate the dominant risk factors. These indications were grouped into the following categories: stable project scope, smooth communication supported by the project management, dedicated, competent and decision-making client team, competent and engaged consultant project manager, schedule and budget consistent with the project scope, use of methodology and procedures, enforced and enabled by the project managers, competent and dedicated consultants. A detailed description is provided for each category.

## Background

Enterprise Systems (ES) are the backbone of the IT infrastructure of most global manufacturing and service enterprises (Muscatello and Chen 2008). Formerly identified with Enterprise Resource Planning (ERP) applications (Davenport 1998; Sedera and Gable 2010), these commercial off-the-shelf (COTS) systems have evolved from plain ERP toward more sophisticated application suites that include ERP, Customer Relationship Management, Business Intelligence, Workflow, Content Management, and other functionalities, which are required to support the information flow and workflow in organisations. Adapting the Klaus et al. (2000) definition, an ES is a standard, customisable application suite that includes integrated business solutions for the major business processes and administrative functions of an enterprise, with the ERP system being the central component of this suite. Enterprise Systems have become a fundamental tool in many industries, i.e., a ‘must-have’ to maintain competitive position on both global and local markets (Al-Mashari et al. 2003; Helo et al. 2008; Beheshti and Beheshti 2010), and these systems continue to attract the attention of researchers (Al-Jabri and Roztocki 2015; Bernroider et al. 2014: Eden et al. 2014; Koch and Mitlöhner 2010; Ram et al. 2014; Trąbka and Soja 2013).

Despite their popularity, the rate of failure in the implementation of ESs is consistently high (Aloini et al. 2007; Beheshti and Beheshti 2010; Wu and Wang 2007).

There is a great deal of discussion concerning the criteria that should be used to designate an implementation a failure (Basten et al. 2011; Basten and Pankratz 2015; Lech 2013a; Yeo 2002), leading to considerable research on implementation success and failure factors regarding both Information Systems in general, and Enterprise Systems in particular. However, there is no doubt that many ES projects still face problems with meeting their original scope, budget, and/or schedule (Belassi and Tukel 1996; Dezdar and Sulaiman 2009; Kappelman et al. 2006; Mu et al. 2015; Nah and Delgado 2006; Nelson 2007; Ram et al. 2014; Somers and Nelson 2001; Schmidt et al. 2001; Soja and Paliwoda-Pękosz 2009; Yeo 2002).

The majority of research studies concentrate on identifying the conditions that are believed to increase the probability of success of a project. These conditions are commonly referred to as critical success factors, or CSFs. The research regarding CSFs has been summarised in the literature review presented in Table 1. For ease of comparison, the CSFs have been mapped with each other, and labelled with common categories, which will be used further in this paper.

**Table 1:** Critical success factors

Dezdar and Sulaiman (2009)	Somers and Nelson (2001)	Common category
95 articles published between 1999 and 2008 in top IS journals	65 research articles and 110 case studies from popular literature, published between 1983 and 2000	
Top management support and commitment	Top management support Use of a steering committee	Top management support
Project champion	A project champion	Project champion
Project management and evaluation	Project management	Project management
Business plan and vision	Clear goals and objectives	Clear goals/scope
Change management programme	Management of expectations Change management	Change management procedures
Use of a consultant	Use of consultants	Use of consultants
Careful selection of ERP software	Careful package selection Choice of architecture	Selection of the proper system
System quality		System quality
Business process reengineering and minimum customisation	Business Process Reengineering Minimal customisation	BPR/Limited customisation of the system
Vendor support	Vendor support Partnership with vendor Use of vendors’ tools	Vendor support
User involvement	Dedicated resources	Team dedication/involvement in the project
ERP team composition, competence and compensation	Project team competence	Expertise of the team
User training and education	User training on software	Team/end-users training
Enterprise-wide communication and cooperation	Interdepartmental cooperation Interdepartmental communication	Effective communication and cooperation
Organisational culture	Education on new business processes	Organisational preparedness
Software analysis, testing and troubleshooting	Data analysis and conversion	Use of methodology

As can be seen from the table above, both literature reviews present lists of success factors that can be easily matched to each other. The matching was done in the table with the use of the ‘common category’, which is self-explanatory in almost all cases. Additional explanation may be needed for the last category, i.e., ‘Use of methodology’. The two success factors grouped under this category are ‘software analysis, testing and troubleshooting’ and ‘data analysis and conversion’. These categories constitute important elements of the implementation methodology but it also has other elements, so a more general category was used as a common denominator.

The critical success factors in Table 1 are high level and are presented from the perspective of the adopting organisation. While they are extremely valuable for the steering committee members, project sponsors, and project managers, a more in-depth, practical list would be useful for the successful execution of the project. For example, a more detailed list would specify exactly what is meant by proper project management, effective communication, or the selection of a proper system. Only a few studies on CSFs have presented an in-depth analysis of sub-factors (e.g., Nah et al. 2003; Ngai et al. 2008; Somers and Nelson 2001).

Several authors took the opposite perspective and attempted to identify the circumstances that cause the projects to fail. If the logical ‘not’ is added in front of each of the success factors presented above, the result might be a list of failure factors or project risks, depending on the moment of observation. If one observes a project that has failed and analyses the causes of this failure, then the result would be a list of failure factors. Such a list can be used to identify the potential problems in the running project, in order to neutralise them before they cause a project to fail. Thus, a project risk can be defined as a ‘potential problem, that has not yet happened, but which could cause some loss or threaten the success of the project if it did.’ (Wiegers 1998). IS literature also includes direct research on project risk/failure factors, some of which is presented in Table 2.

**Table 2:** Project risks/failure factors

	Nelson (2007) Project mistakes	Kappelman et al. (2006) Early warning signs	Yeo (2002) Critical failure factors	Schmidt et al. (2001) Project risks
1.	Poor estimation and/or scheduling	Lack of top management support	Underestimate of timeline	Lack of top management commitment to the project
2.	Ineffective stakeholder management	Weak project manager	Weak definitions of requirements and scope	Misunderstanding the user requirements
3.	Insufficient risk management	No stakeholder involvement and/or participation	Inadequate project risk analysis	Not managing change properly
4.	Insufficient planning	Weak commitment of project team	Incorrect assumptions regarding risk analysis	Failure to gain user commitment
5.	Short-changed quality assurance	Team members lack requisite knowledge and/or skills	Ambiguous business needs and unclear vision	Lack of adequate user involvement
6.	Weak personnel and/or team issues	Subject matter experts are overscheduled	Lack user involvement and inputs from the onset	Conflict between user departments
7.	Insufficient project sponsorship	Lack of documented requirements and/or success criteria	Top down management style	Changing scope and objectives
8.	Poor determination of requirements	No change control process (change management)	Poor internal communication	Number of organisational units involved
9.	Inattention to politics	Ineffective schedule planning and/or management	Absence of an influential champion and a change agent	Failure to manage end-user expectations
10.	Lack of user involvement	Communication breakdown among stakeholders	Reactive and not pro-active in dealing with problems	Unclear/misunderstood scope and objectives
11.	Unrealistic expectations	Resources assigned to a higher priority project	Consultant/vendor underestimated the project scope and complexity	Improper definitions of roles and responsibilities
12.	Undermined motivation	No business case for the project	Incomplete specifications when project started	Lack of frozen requirements
13.	Contractor failure		Inappropriate choice of software	Introduction of new technology
14.	Scope creep		Changes in design specifications late the project	Lack of effective project management skills
15.	Wishful thinking		Involve high degree of customisation in application	Lack of effective project management methodology
16.				Lack of required team knowledge/skills
17.				Insufficient/inappropriate staffing

Although the level of detail and the exact naming of particular risk factors differ across the literature, it can be assumed that there is agreement between the researchers on which risk factors negatively affect the ES projects.

However the answers to the questions ‘Why do these failure factors emerge in most of the ES projects?’ and ‘What remedies can be applied to compromise the most prominent risk factors?’ are not usually answered. Furthermore, all of the research cited above presents the risk factors from the perspective of an adopting organisation, while a typical project landscape consists of three parties (Haines and Goodhue 2003): the adopting organisation (implementer, client), the system vendor, and the consulting enterprise (consultant) that helps the adopting organisation to implement the system successfully. For a description of the role of consultants in the implementation of an Enterprise System project, see Lech (2013b). Therefore, it is a promising research direction to investigate the failure factors in more detail and to present a perspective other than that of the adopting organisation. This study investigated the causes of the dominant risk factors and proposed remedies for those risk factors from the perspective of the implementation consultants involved in ES projects.

## Results and discussion

The earlier study, which was performed in 2012 among fourteen ES consultants, representing 174 projects, resulted in the ranking of the most influential risk factors presented in Table 3.

**Table 3:** Ranking of the most influential risk factors according to consultants

Failure/risk factor
1. Unclear/changing goals/scope/requirements
2. Communication problems
3. Lack of client team dedication to/involvement in the project
4. Poor project management
5. Lack of change management procedures
6. Poor planning/estimation/scheduling
7. Improper/insufficient client team expertise
8. Consultants overscheduled
9. Lack of/insufficient resources/consultants
10. Lack of decision-making capabilities in the client team

The above list was a starting point for the study presented in this paper. The aim of the study was to investigate the causes of those dominant risk factors and propose remedies for them from the perspective of implementation consultants involved in ES projects. The results of the research are presented in the following sections. The methodology of the research is presented in the “Methods” section at the end of the paper.

### Stable project scope

‘Unclear/changing goals/scope/requirements’ was indicated as the most important risk factor. The reasons, according to the consultants, are the lack of client team dedication to the project in its early phases when the project scope included in the contract is being detailed and transformed into the system design, improper change management and a lack of system knowledge in the client team. The consultants did not indicate errors in the project scoping/bidding phase, most probably because they are responsible for project execution, not for planning and preparation, and they treat the project scope, schedule, and budget as imposed. The planning of an ES project, especially regarding budget and schedule, is an understudied research area. A recent literature review on ES, made by Eden et al. (2014), does not include any papers covering that topic. More research is available on ERP Requirements Engineering. A summary of this research is presented in Daneva and Wieringa (2010).

The consultants also indicated other factors that determine the stability of the scope of the project during execution. One of the initial phases of a project (after project planning) is the design (or blueprint) phase. During this phase, abstract requirements that are included in the project scope are transformed into detailed specifications, describing how these requirements will be included in the new system. Careful execution of this step significantly increases the probability that the system will meet the client company’s needs. Therefore, active participation of the client project team, including the participation of its business domain experts, is crucial in this phase, as these are the people who can detail the requirements and verify that the solutions proposed by the consultants are in line with these requirements. Achieving this goal is much easier if the client project team is familiar with the system being implemented. As this is rarely the case, the client project team should go through preliminary training, during which the basics of the system are presented. Whenever possible, the design process should be supplemented by demonstrations of the functionality of the system. This will help the members of the client project team to visualise the solutions included in the system design (Blueprint) document. According to the consultants who participated in the interviews, this is only possible in specific situations, such as when the functionality to be presented does not require substantial system configuration.

Another factor that helps to keep the scope of the project stable is change management. According to the consultants interviewed, this means resistance to uncontrolled changes of scope by the consultant project manager, as well as eagerness to adapt the business processes to the system functionality of the client organisation, and management of the expectations of the client’s management. As consultants are only one part of the project team, these findings should all be compared with the findings of the studies that considered the point-of-view of the client. Consultants are motivated to accomplish the project within a given scope, budget and schedule. However, if the scope of the project is not determined with enough care, or the business environment of the client organisation has changed during the course of the project, it may be necessary to change the scope and adjust the schedule and budget accordingly (Lech 2013a). On the other hand, the consultants also reported that in some projects, the scope increases drastically due to the incorporation of requirements that are not necessarily related to the company’s core business, but that reflect either current business practices of particular users or that are only included to make the users’ lives easier. Such ‘nice-to-have’ requirements should be carefully evaluated by the client management, who should compare their value added with the resulting increase in budget and time, as well as with the potential increase in future costs related to the maintenance of a highly customised system (e.g., problems during upgrades).

In summary, control of the scope of the project can be achieved through:

1. A careful definition of the scope during project preparation/bidding;
2. Involvement of the client project team in the early phases of the project;
3. Careful change management and expectations management by avoiding unnecessary system customisation, by adjusting the business processes to the system functionality in non-critical areas, and by resistance to unnecessary changes from the project management.

These recommendations address the risk factors: ‘Unclear/changing goals/scope/requirements’ and ‘Poor planning/estimation/scheduling’.

### Smooth communication supported by the project management

‘Communication problems’ ranked second in the list of risk factors, according to consultants. This indicates that, first, communication among the project participants is extremely important and second, that consultants have noticed significant flaws in communication during projects in which they have participated. During the in-depth interviews, the consultants identified causes for communication problems, and suggested remedies for them. The first problem, highlighted by the consultants, is communication ambiguity on the client side. Consultants reported that in some projects, they faced problems with finding the right person with whom to communicate. The lack of a clear communication path results in difficulties with the clarification of questions and with obtaining decisions regarding the required functionality of the system. In the best-case scenario, after addressing a question to all the client team members, they obtained the answer from one of them (which caused unproductivity on the client side, because people who were not responsible for or not able to respond to the question were forced to deal with it). In the worst-case scenario (and the most common one), the question remained unanswered, as no one on the client side felt responsible to react. The remedy to this problem is a single-point-of-contact for each of the consultants, which should be a key-user who is responsible for a given functional area. A communication map, indicating contact persons for each functional area should be clearly defined in the project procedures, and the project managers should be required to enforce it.

Another instance of ambiguity in communication on the client side occurs when different stakeholders pose conflicting requirements. This situation is related to a lack of clear vision regarding the aims and goals of the project, and to politics within the client organisation, especially between different departments. According to the consultants, the remedy for this problem is the presence of a strong project leader on the client side. He/she may also be the project manager or may cooperate closely with the project management team. His/her responsibility is to work out compromises when conflicting requirements occur, or, if this is not possible, to make binding decisions on which of the conflicting requirements is going to be implemented in the system. If the conflict cannot be resolved by the project management team and the project leader, it is the role of the Steering Committee to make a final decision. The consultants emphasised the role of the Steering Committee in solving the major problems in the project.

For this to happen, the conflict between the requirements has to be identified first, which, according to the respondents, should be done during status/integration meetings. All of the respondents pointed towards the importance of status/integration meetings as the main tool for facilitating the communication within and between the functional teams. According to them, status meetings within a team should take place every week, while integration meetings, with the presence of all functional teams, should take place no less frequently than every 2 weeks. These meetings should be structured and facilitated by the project management team. All the decisions made during the meetings should be documented and approved by the team leaders, and appropriate procedures to deal with the identified problems and risks should be applied.

Communication issues between the client and consultant arise due to the aforementioned problems on the client side, but may also be caused by a culture gap, the consultants’ lack of soft skills, or improper task management by the consultants. The culture gap is a phenomena extensively described in the literature and refers to a situation when the two (or more) parties (here, the consultant and the client) cannot communicate properly due to different cultural and professional backgrounds. The classical understanding of a culture gap has assumed that IT people cannot communicate properly with business people due to the lack of knowledge of each other’s domain of expertise. To avoid this situation, the consultants should be the domain experts in their respective functional areas. As this was the case for the interviewees in this research, this classical culture gap was not reported as a failure/risk factor. However, the consultants reported communication problems due to the clients’ lack of system knowledge. This is one of the risk factors that is most difficult to mitigate, as it is hard, if not impossible, to expect the clients to be experts in systems that are new to them. The purpose of involving the consultants in a project is to benefit from their system knowledge, which can then be transferred gradually to the client. During the design phase, however, the client does not usually possess enough knowledge to verify the proposals made by the consultants in an informed way. A partial remedy to this problem is initial training for the client team. However, as ES systems are highly configurable and usually also require customisation, this initial training cannot reflect the configuration/customisation of the target system. Training can be carried out on demo/training systems, but these may differ significantly from the system after it has been configured and customised during the project. Therefore, the aim of this training can only be to familiarise the client with the nomenclature and general logic of the system. Another way of decreasing the culture gap on the client side is to perform demonstrations of parts of the system whenever possible, during the design and early implementation phases. However, this is only possible for those parts of the system that do not require substantial configuration and customisation.

The consultants also indicated that some of the reasons for the communication problems lay solely on their side. These are a lack of soft skills of some of the consultants and improper task management. The lack of soft skills may cause a breakdown in communication between the consultant and his/her counterparts on the client side. The most obvious solution, which is often not easy to achieve, is training the consultants in soft skills. This requires time and has to be done before the project. If communication problems arise during the project, they should be monitored and resolved by the consultant project manager. In the most extreme situations, according to the respondents, the project manager should attend the meetings of a team that is facing communication problems, in order to actively animate the discussions and to help find solutions for problems that have arisen. ‘Improper task management’ involves imprecise statements regarding what is required from the client, a lack of assignment of a responsible person on the client side, and a lack of deadlines. These problems should be resolved by project methodology, part of which is the documentation of every meeting with the client. The documentation format should enforce the preparation of a task list, including the person/people responsible and the deadline for each task. The project manager should control the documentation on a regular basis and enforce the proper documentation of the findings and the tasks.

Communication problems can also occur within the consulting team, between different functional consultants, and between the consultants and their project managers. In fact, these were the communication problems that were most commonly mentioned by the respondents. The causes for communication problems within the consulting team include the lack of consultants’ soft/communication skills, the consultants’ encapsulation within their functional area, and the lack of or improper execution of communication procedures.

These three causes tend to interfere with each other, because the project activities are usually performed on a daily basis within the teams that cover one functional area (e.g., financials, inbound logistics and inventory, outbound logistics and sales, production planning, and execution) and both the consultants and their counterparts in the client team tend to focus on the problems that arise within their respective functional areas. If the consultants lack soft/communication skills, they may tend to avoid confronting the other teams, so it is difficult for them to identify integration points and resolve possible inconsistencies. Also, less experienced consultants may not be aware of the ways that the configuration of their respective functional area may interfere with the configuration in other areas. If, in addition, the communication procedures do not force them to discuss and reconcile these integration issues, the resulting system may be inconsistent, i.e., the fulfilment of the requirements in one functional area may preclude the fulfilment of the requirements in another area.

The remedies for these problems are the inclusion of training of the consultants in soft skills in their career paths, and the careful preparation and execution of the communication procedures during the project. Once again, the key item in communication procedures is the careful preparation and execution of the status/integration meetings. The respondents emphasised the need for the project managers to play an active role in these meetings. They should not only gather information from the teams, but should also actively seek to identify possible integration problems as well as to participate in their solutions. For this to happen, the project managers need to have both company knowledge (the client project manager) and system knowledge (the consultant project manager). The results of these meetings should be documented, approved by the participants, and treated as binding after having been approved.

In summary, to assure proper communication, the following conditions must be met:

1. Consultants must possess knowledge in their functional areas, in order to speak the same language as the clients and understand their requirements;
2. Consultants should possess strong communication skills;
3. A clear communication map and communication procedures must be developed and consequently applied;
4. The client team should learn about the system through initial training and through presentations of the functionality of the system in the early phases of the project;
5. A strong project leader should be present on the client side to resolve problems with conflicting requirements;
6. Communication should be monitored and animated by the project managers on a regular basis;
7. All findings should be documented and approved both by the client and the consultant;
8. All tasks should be documented and have a person responsible and deadline assigned;
9. And, most importantly—status/integration meetings must be held frequently, with project managers playing an active role in the identification and solution of the issues that arise.

These recommendations address the risk factors: ‘Communication problems and Lack of change management procedures’.

### Dedicated, competent client team with decision-making capabilities

‘Lack of client team dedication’, ‘Improper/insufficient team expertise’ and/or ‘Lack of decision-making capabilities’ were the three risk factors attributed to the people involved in the project from the client’s side. Because the consultants are outside the implementing organisations, they could not identify causes for these problems, nor give specific advice on how to achieve the desired characteristics of the members of the client team. However, they did present a normative stance on what this team should represent.

1. The client team should consist of the domain experts in the areas involved in the implementation. The most competent people should be dedicated to the project as key-users.
2. The key-users should dedicate at least 50 % of their time to the project in the design and testing phases. Involvement in the realisation phase may be lower, but the more that the key-users are involved in the realisation phase, the less knowledge transfer is needed before and just after the ‘project go-live’.
3. The key-users should have decision-making capability, i.e., they either should be entitled to make business decisions regarding business process change, and design decisions regarding system functionality if alternatives exist, or else, they should have a short path to the decision makers to enable fast decision making.

Another issue that the client company may face is the difficulty of achieving points 1 and 2 at the same time. The most competent domain experts are usually the most overloaded people in the company, which makes it extremely difficult to take them away from their daily activities so that they can devote half of their time to the project. Consultants identified three approaches for the client companies to solve the issue of overscheduling. The first is to have the domain experts, being the key-users, work overtime for the duration of the project, which requires extremely high motivation. Another approach is to nominate people with less experience but more time as key-users and to grant them access to the domain experts when needed. This resolves overload of the experts but poses additional communication issues between the non-expert key-users and the domain experts. The third solution identified is to hire part-time, additional staff to perform the daily activities, so that the domain experts can concentrate on the project.

These recommendations address the risk factors: ‘Lack of client team dedication’, ‘Improper/insufficient team expertise’, and/or ‘Lack of decision-making capabilities’.

### Competent and engaged consultant project manager

The role of a consultant project manager in facilitating (or hindering) the success of a project is manifested by the many indications for improvement in the execution of projects that were mentioned above. Poor project management also ranked fourth in the list of the project risk factors. The respondents clearly indicated the characteristics that distinguish a good project manager from a poor one. Besides the general project management skills necessary to manage a project’s scope, budget, and schedule, a good project manager should also:

1. Possess the system knowledge and experience from other projects to be able to identify possible risks and problems, and find solutions;
2. Actively prioritise and coordinate tasks in and between the projects—he/she should be aware of the complexity and interdependencies of the tasks;
3. Carefully listen to the team, give support and be open to suggestions from the team;
4. Support the consultants in front of the client—play ‘bad cop’ so that the consultants can maintain a good relationship with the client team;
5. Actively participate in the identification of problems/risks and their solutions;
6. Be assertive in front of the client to defend the project scope, budget, and schedule;
7. Organise and actively participate in the status/integration meetings;
8. Enable and streamline the communication in and between the teams;
9. Implement and actively enforce the usage of a project methodology and procedures.

As one of the respondents recapitulated, “There are bad project managers who just administer their MS Project or Excel files, prepare a task list, and expect the tasks to be done somehow, without their involvement. And there are good project managers who just make a project happen”.

These recommendations address the risk factors: ‘Poor project management’ and ‘Lack of change management procedures’.

### Schedule and budget consistent with the project scope

Poor planning/estimation/scheduling, together with ‘Lack of change management procedures’ were the two risk factors that refer to the inconsistencies between the scope, and the other two ‘iron triangle’ project parameters, i.e., budget and schedule.

Because consultants have an operational role in the project, being responsible for its execution, and not for its planning and preparation, they tend to treat the project scope, schedule, and budget as imposed. Therefore, in the interviews, they did not give any specific recommendations on how the bidding and project preparation phases should be executed in order to determine a feasible scope, together with a corresponding budget and schedule. However, they did point out some of the causes of inconsistency between the scope, schedule, and budget. The first is unrealistic expectations of the client due to a lack of knowledge about the complexity of the project. The second is acceptance of these unrealistic expectations by the consulting enterprise because of strong competition in the market, reasoning that ‘if we do not take this project, someone else will’ and ‘after we start the project, we will be able to renegotiate the contract once the customer realises that the initial assumptions were not feasible’. The remedy for this is a careful bidding process, including a definition of the scope of the project in a structured and methodological way, and also the inclusion of a realistic budget and schedule, according to that scope. The client should also learn about the specifics of the proposed system and the related complexity of the project before starting the implementation. However, this study did not reveal any particular indications on how this should be achieved. During the execution of the project, it is crucial to apply a proper, methodological procedure for change management. The project manager should resist any change of scope that is not followed by respective adjustments to the budget and the schedule.

These recommendations address the risk factors: ‘Poor planning/estimation/scheduling’ and ‘Lack of change management procedures’.

### Use of methodology and procedures, enforced and enabled by the project managers

The consultants also pointed out that improper application of project management methodology may hinder the success of a project. According to them, the problem in the projects in which they had participated was not the absence of methodology, as the methodology was always in place, but deficiencies in the execution of the methodology during the project. The main causes for this were poor project management/manager, and time pressure. The importance of the project manager’s active role was discussed in the previous sections. The project manager must not only introduce the methodology at the project preparation phase, but more importantly, must also control and, if necessary, enforce its execution during the project. Time pressure, which most projects face, makes some of the project participants (both consultants and clients) treat the methodology requirements, such as proper documentation, testing (especially regression testing), and integration meetings as an unnecessary burden that drags them away from the ‘real work’ on system configuration and customisation. This results in a ‘firefighting syndrome’, in which changes to the system are made without proper documentation and testing, and without evaluation of their impact on other functionality. The respondents found this situation extremely dangerous and advised that no part of the methodology should ever be omitted. In particular, they mentioned the following activities as important for the success of a project:

1. A timely execution of status/integration meetings;
2. The careful documentation of all findings;
3. A precise definition of tasks, with the people responsible and the deadlines assigned to each of them;
4. A methodological approach to change and risk management;
5. The careful preparation and execution of the test phase:
  - Careful preparation of the test scripts;
  - Proper scheduling of the tests, with enough time granted for corrections between the test runs;
6. Clear responsibility divided between the project participants.

Once again, it is important to emphasise the role of a project manager in enforcing the application of the methodology during the project.

The use of methodology helps to minimise the following risk factors: ‘Unclear/changing goals/scope/requirements’, ‘Lack of change management procedures’, ‘Poor project management’.

### Competent and dedicated consultants

The consultants also complained about some issues regarding their work in the projects, which resulted in the inclusion of the risk factors into the ranking: ‘Consultants overscheduled’ and ‘Lack of/insufficient resources/consultants’.

They stated that they are sometimes assigned to tasks that exceed their competence. Often they are also overloaded, working on several projects at the same time and also supporting the sales processes for new customers. It also happens that the project staffing does not always correspond to the workload. The only remedy that the respondents saw for this overload is a full-time contract, in which a customer pays for 100 % of the consultant’s time. This prevents the consulting enterprise from assigning him/her to another project at the same time. However, this approach may raise the cost of the implementation significantly. Regarding the issue of insufficient competence, the remedy for the customer is to carefully examine the CV’s of the consultants who are supposed to execute a project, interview the consultants, and check their references. It is also a good practice to ensure in the contract that the consultant presented during the project preparation will be actually executing the project.

The summary of recommendations for improvement of in the execution of projects from the consultant’s perspective is presented in Table 4.

**Table 4:** A summary of the recommendations for improvement of the execution of projects from the consultant’s perspective

Indication	Activities	Risk factor addressed
Keep the project scope stable	Perform careful project scoping during the bidding process Assure client team involvement, especially during the design phase Train the client team in the system before or at the beginning of the project Prototype during the design phase when possible (so that no substantial configuration and no customisation are needed) Adjust the business processes to the system functionality in areas that do not harm competitiveness Manage client expectations: include ‘must-haves’, exclude ‘nice-to-haves’	Unclear/changing goals/scope/requirements Poor planning/estimation/scheduling
Introduce and maintain smooth communication supported by the project management	Establish and hold periodic status/integration meetings, empowered and animated by the project managers Prepare a clear communication map with single points of contact Introduce and enforce the usage of communication procedures Document all findings and tasks, and assign responsibility and deadlines to the tasks Manage conflicting requirements by appointing a strong project leader (champion) on the client side and involve the Steering Committee in problem solving if necessary Reduce the culture gap by appointing consultants which have domain expertise and strong soft skills training the client team in the system involving prototyping and functionality presentations in the design phase whenever possible Make the enablement of communication one of the main tasks for the project managers	Communication problems Lack of change management procedures
Assure dedicated, competent, client team with decision-making capabilities	Assign domain experts to the team as key-users or grant the key-users constant access to the domain experts Grant the key-users decision-making capability or grant them constant and quick access to the decision makers Grant the key-users time to participate in the project motivate them to work over-time; or nominate less experienced staff as key-users but grant them constant access to experts; or hire temporary employees to perform the daily activities of the key users	Lack of client team dedication to/involvement in the project Improper/insufficient client team expertise Lack of decision-making capabilities in the client team
Appoint a competent and engaged consultant project manager	The consultant project manager should possess the system knowledge and experience from other projects to be able to identify possible risks and problems, and find solutions actively prioritise and coordinate tasks in and between the projects—he/she should be aware of the task complexity and interdependencies carefully listen to the team, give support and be open to suggestions from the team support the consultants in front of the client—play ‘bad cop’ so that the consultants can maintain a good relationship with the client team actively participate in the identification and solving of problems/risks be assertive in front of the client in defending the project scope, budget, and schedule organise and actively participate in the status/integration meetings enable and streamline the communication in and between the teams implement and actively enforce the usage of a project methodology and procedures	Poor project management Lack of change management procedures
Keep the budget and schedule consistent with the scope	Perform careful project estimation during the bidding process Avoid wishful thinking—evaluate the impact of changes in scope on budget and schedule rationally Apply a methodological change management process Resist any change in scope without schedule and budget adjustments	Poor planning/estimation/scheduling Lack of change management procedures
Enforce the usage of the methodology and procedures	Execute timely status/integration meetings Carefully document all findings Precisely define the tasks, with people responsible and deadlines assigned to them Apply a methodological approach to change and risk management Carefully prepare and execution of the test phase carefully prepare the test scripts grant enough time for corrections between the test sessions Define and enforce clear responsibility split between the project participants	Unclear/changing goals/scope/requirements Lack of change management procedures Poor project management
Assure that the consultants are competent and dedicated to the project	As a client check the consultants CVs and references assure that the consultants’ expected workload is in line with the project scope assure that the consultants presented to you will be actually executing the project	Consultants overscheduled Lack of/insufficient resources/consultants

## Conclusions

The purpose of this study was to investigate the causes of the dominant risk factors affecting the implementation of Enterprise System projects and propose remedies for those risk factors from the perspective of the implementation consultants.

The research presented in this paper took a different perspective on project risk factors than that of most of the existing literature. Instead of looking at ES projects from the ‘general’ perspective of top management and project management, this study analysed projects from the operational perspective of one of the two parties that actually implement the project—the consultants. The results of this research should be treated as an addition to, not a contradiction of, the existing body of knowledge. The limitation of this study is that it examined only one of the two parties involved in the operational execution of the projects. The results should be compared with research that examines the point-of-view of the client team, which is a promising, and unexplored research area, because most of the research involving users concentrates on the phenomenon of user acceptance. The main contribution of this study is that it offers viable indications on how to overcome the dominant risk factors that negatively affect the everyday work during the implementation of an Enterprise System project. The careful consideration of the causes and the implementation of remedies for the risk factors brought to light by this research should help Enterprise Systems projects to proceed more smoothly and with a greater probability of success.

## Methods

As discussed earlier in this paper, most of the existing research on the risk factors in ES implementations was performed from the perspective of the adopting organisation, and little is known about the risk factors from the perspective of the consultants. According to Creswell (2009, p. 18), “if a concept or phenomenon needs to be understood because little research has been performed on it, then it merits a qualitative approach”. Therefore, a qualitative research strategy was chosen for this study. The following research questions were posited.

Q1: What are the causes for the risk factors that can occur during the implementation of an Enterprise System project?

Q2: What remedies can be applied to mitigate the risk factors during the implementation of an Enterprise System project?

Qualitative research calls for multiple data sources and data triangulation to increase the reliability of the study (Yin 2003, p. 116). To meet this requirement, the following data sources were used.

1. E-mail interviews were carried out with fourteen ES consultants, during which they were asked to name any number of risk factors that, in their opinion, negatively influence their ability to accomplish the project with success. The interviews were performed as a part of another study on project failure, but as a side effect, the informants also gave substantial insight into the causes of the project risk factors, which motivated the follow-up study, presented in this paper.
2. Semi-structured face-to-face interviews were carried out with four respondents who were active ES consultants and also had experience as project managers, with seniority ranging from 5 to 15 years, and with accumulated knowledge from 52 full-time implementation projects. The informants were presented with a the list of risk factors depicted in Table 3, and were asked to elaborate on the causes for these risk factors and to suggest remedies that should be implemented to avoid or mitigate them.
3. Participant observation from six projects in which the author was actively involved as a consultant and/or project manager was included in the data. This data source was used as a triangulation source for interviews with the consultants, for the purpose of double-checking the data saturation in the interviews.

The following research procedure was applied to the above data sources.

1. The responses from the e-mail interviews were coded with the use of MAXQDA qualitative research software. The coding procedure was used to answer Q1, i.e., finding the phrases that indicate causes for the risk factors to occur in the project.
2. During the semi-structured face-to-face interviews, the informants were explicitly asked to define the causes for the risk factors to occur in the project and to propose remedies that may be used to mitigate those risk factors. The interviews were recorded, transcribed, and coded with the use of MAXQDA software. The answers regarding Q1 were triangulated with the results from Step 1. The answers regarding Q2 were open coded and were subject to triangulation during Step 3.
3. The results of the open coding with regard to risk remedies were triangulated with the use of the field notes from participant observation from six projects.

The results were summarized in a form of a narrative and presented in the above sections of this paper.
